# Supplementary material for: NR2F2 regulation of interstitial cell fate in the embryonic mouse testis and its impact on differences of sex development
Source: Nat Commun. 2025 Apr 29;16:3987. doi: 10.1038/s41467-025-59183-6 (PMC12038043; doi:10.1038/s41467-025-59183-6)
Supplement: Supplementary file 1 — Supplementary Information [file 41467_2025_59183_MOESM1_ESM.pdf]

Supplementary Figure 1

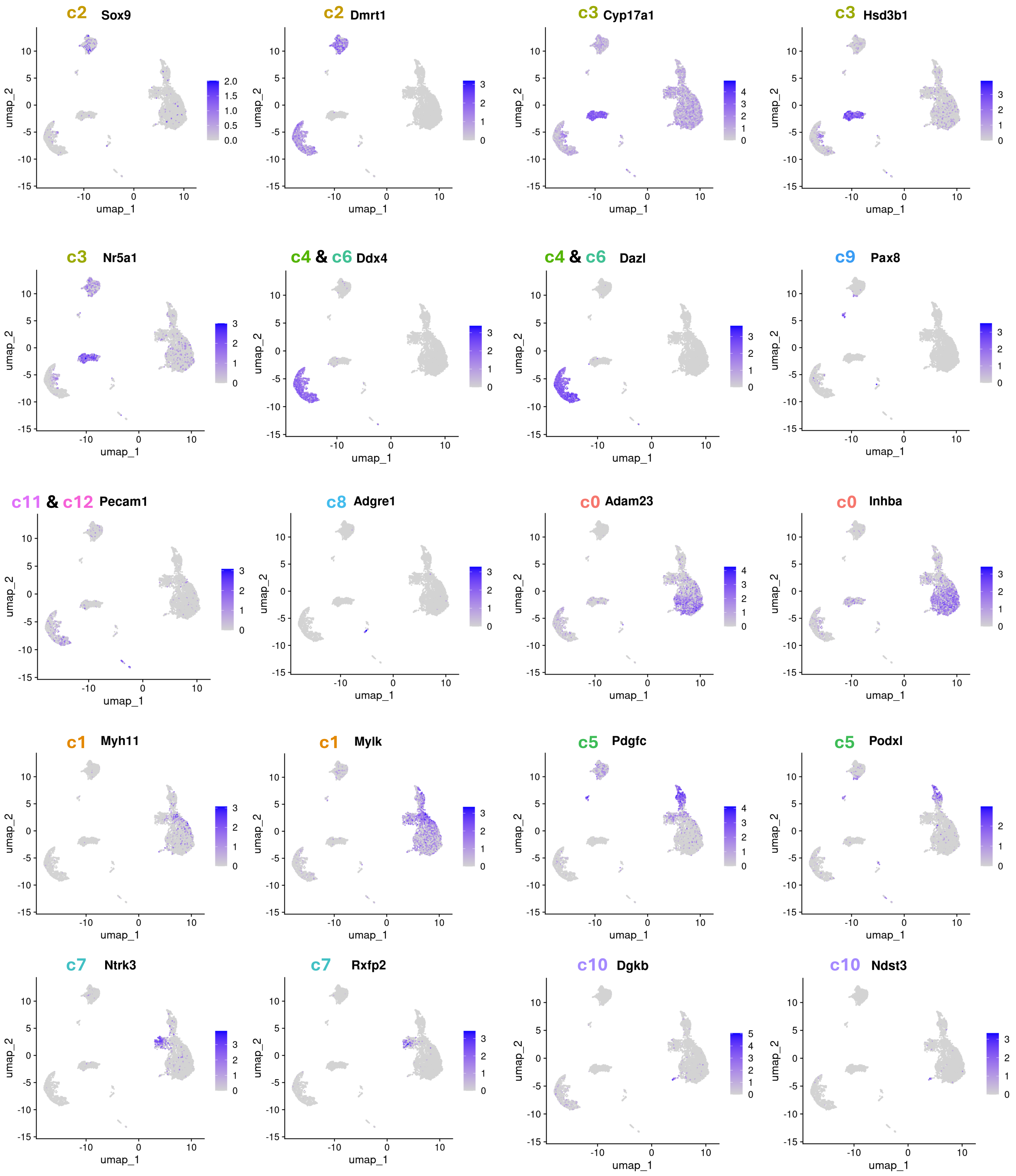

Supplementary Fig. 1: Cluster specific gene expression. Feature plots of the normalized expression of cluster specific genes on the multiomic UMAP visualization of E14.5 testes.

Supplementary Figure 2

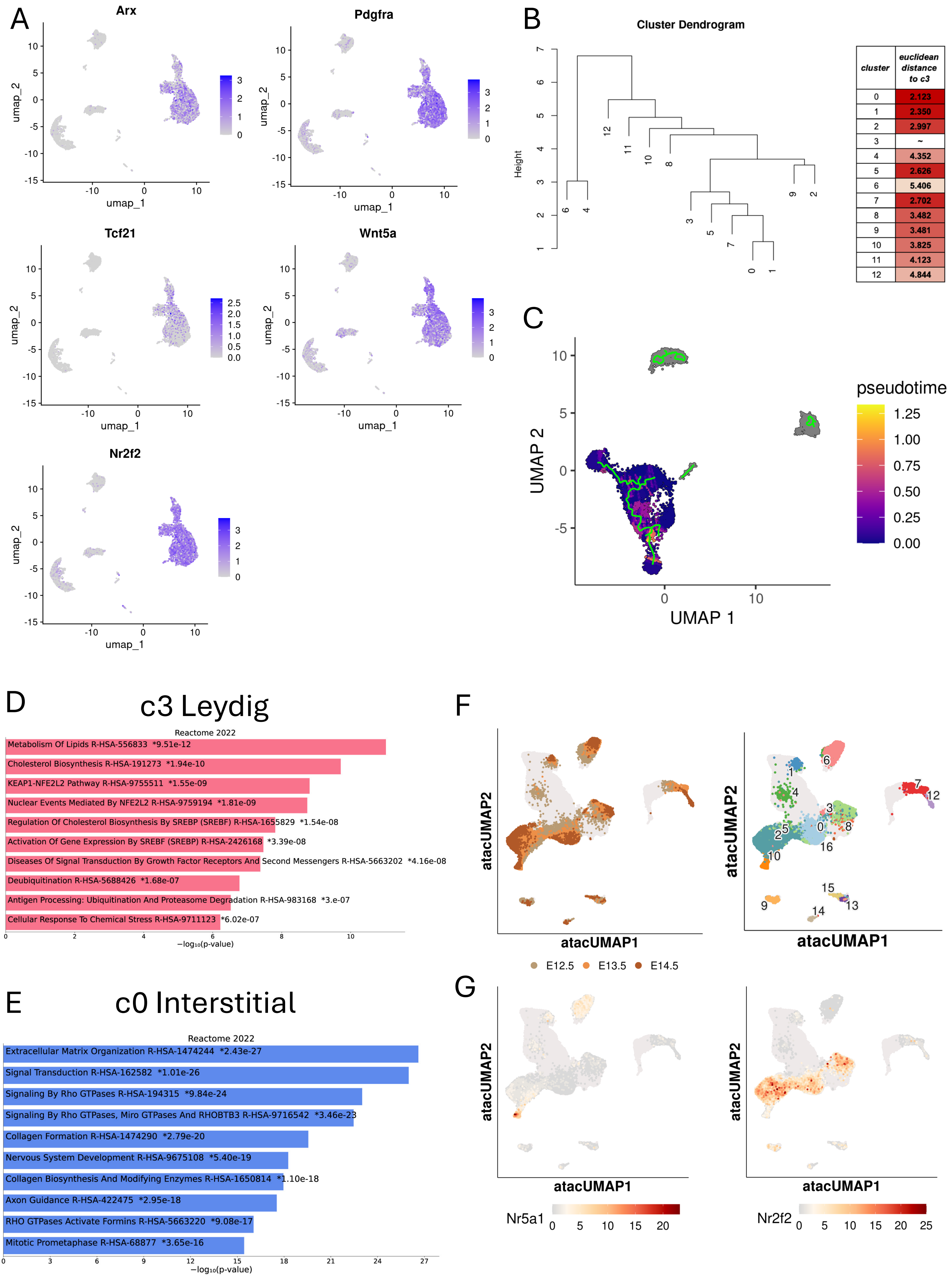

Supplementary Fig. 2: Transcriptomic characterization of Leydig cells and Leydig progenitors. (A) Feature plots of the normalized expression of interstitial genes on the multiomic UMAP visualization of E14.5 testes. (B) Cluster dendrogram based on Euclidean distance between cluster averages at the 2000 most variable snATAC-seq peaks. (C) Monocle3 trajectory analysis based on snATAC-seq data. The pseudotime color scale refers to the partition including clusters c3 (with c0 defined as the root); cells in other partitions are shown in gray. Reported trajectories from all partitions are shown in green. Pathway analysis (Reactome 2022) of c3 (D) or c0 (E) DEGs. * indicates adjusted p-value <0.05. (F) UMAP of the single-nucleus ATAC-seq data from E12.5, E13.5 and E14.5 gonads, color-coded based on the testicular stage origin and cell clusters. (G) Feature plots of the normalized expression of *Nr2f2* and *Nr5a1* on the E12.5-E14.5 multiomic UMAP.

Supplementary Figure 3

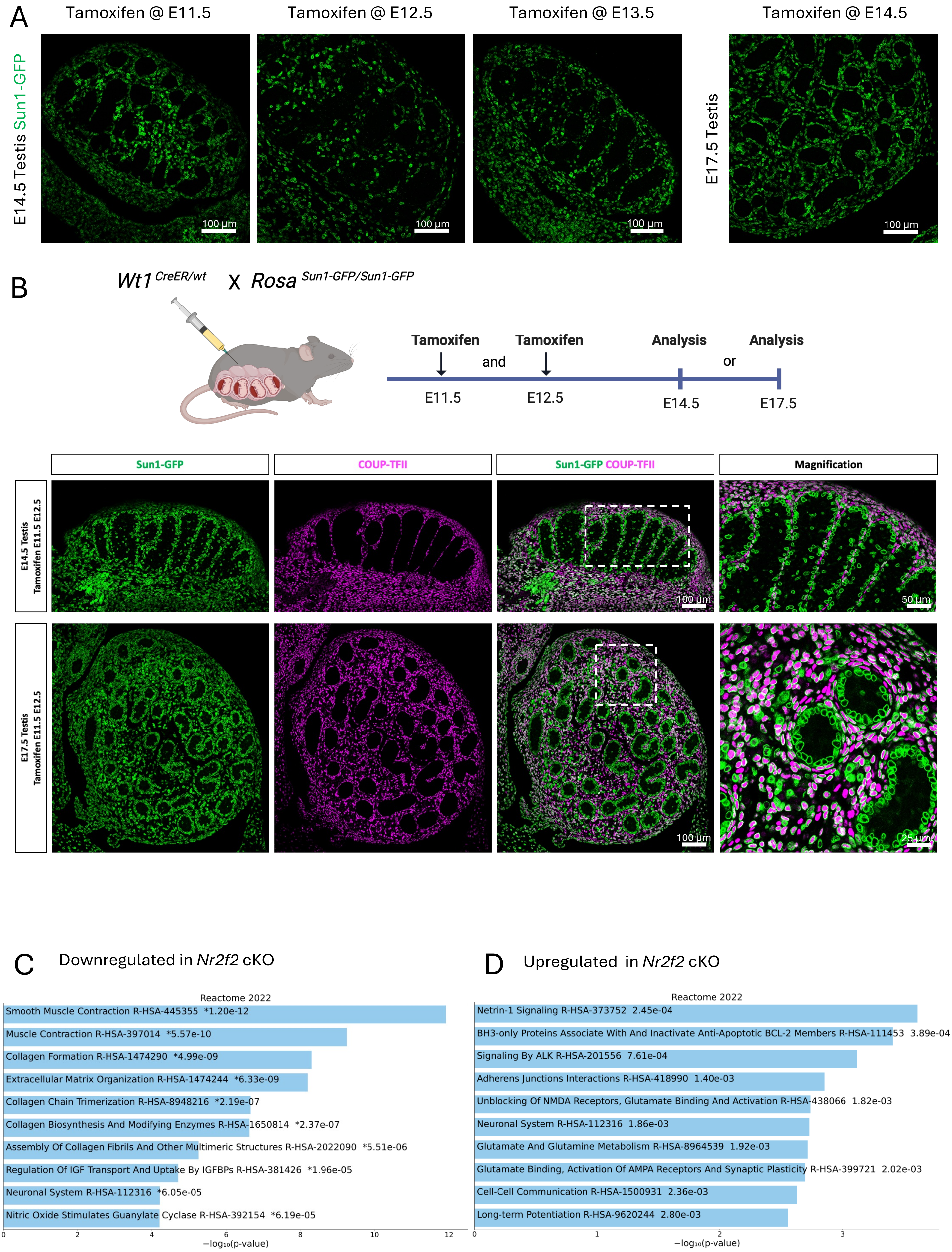

Supplementary Fig. 3: Lineage tracing of *Nr2f2* and *Wt1* positive cells in the fetal testis. (A) GFP only channel of the lineage tracing experiment of the fetal testis-derived *Nr2f2*+ cells in the *Nr2f2*-CreER; *CAG-Sun1/sfGFP* embryos. Cre activity was induced by tamoxifen administration at E11.5, E12.5 E13.5 or E14.5 and immunofluorescence was performed on E14.5 or E17.5 testes, for GFP (Green). (B) Lineage tracing of the fetal testis-derived Wt1+ cells in the *Wt1*-CreER; *CAG-Sun1/sfGFP* embryos was induced by tamoxifen administration at E11.5 and E12.5. Immunofluorescence for GFP (Green) and NR2F2 (Magenta) was performed on E14.5 or E17.5 testes. White dashed box indicates the magnified area. Created in BioRender. Yao, H. (2025) https://BioRender.com/ygb4q06. Pathway analysis (Reactome 2022) of all the downregulated (C) or all the upregulated (D) genes in the knockout testes vs the control. * indicates adjusted p-value <0.05.

Supplementary Figure 4

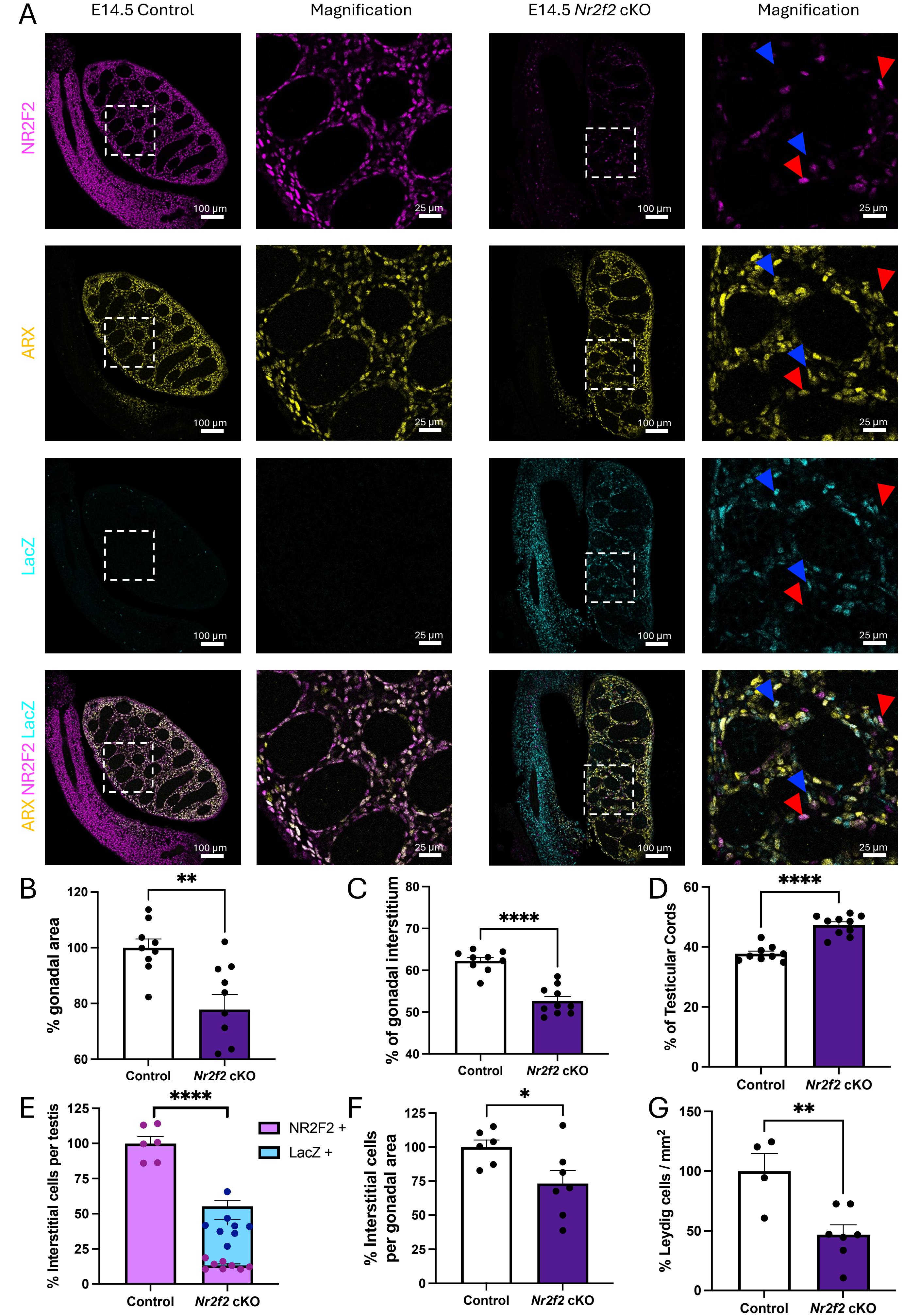

Supplementary Fig. 4: Characterization of the *Nr2f2* knockout testicular phenotype. (A) Immunofluorescence for NR2F2 (magenta), LacZ (cyan) and ARX (yellow) on E14.5 control or *Nr2f2* cKO testes. White dashed box indicates the magnified area. Red arrowheads indicate ARX+ NR2F2 positive/LacZ negative cells. Blue arrowheads indicate ARX+ NR2F2 negative/LacZ positive cells. Quantification of the % of gonadal area (B), % interstitial area (C) and % of testicular cord area (D) in E14.5 control and *Nr2f2* cKO gonads. Bars represent mean±s.e.m., n=9 control and n=10 *Nr2f2* cKO. (E) Quantification of the % of interstitial cells per testis, in E14.5 control and *Nr2f2* cKO gonads, normalized to the control, color-coded by the expression of NR2F2 or LacZ. Bars represent mean±s.e.m., n=6 control and n=8 *Nr2f2* cKO. (F) Quantification of the % of interstitial cells per gonadal area, relative to the control testis. Bars represent mean±s.e.m., n=6 control and n=7 *Nr2f2* cKO. (G) Quantification of the % of fetal Leydig cells per gonadal area, relative to the control testis. Bars represent mean±s.e.m., n=4 control and n=7 *Nr2f2* cKO. Unpaired two-tailed t-test. *P<0.05; **P<0.01; ****P<0.001.

Supplementary Figure 5

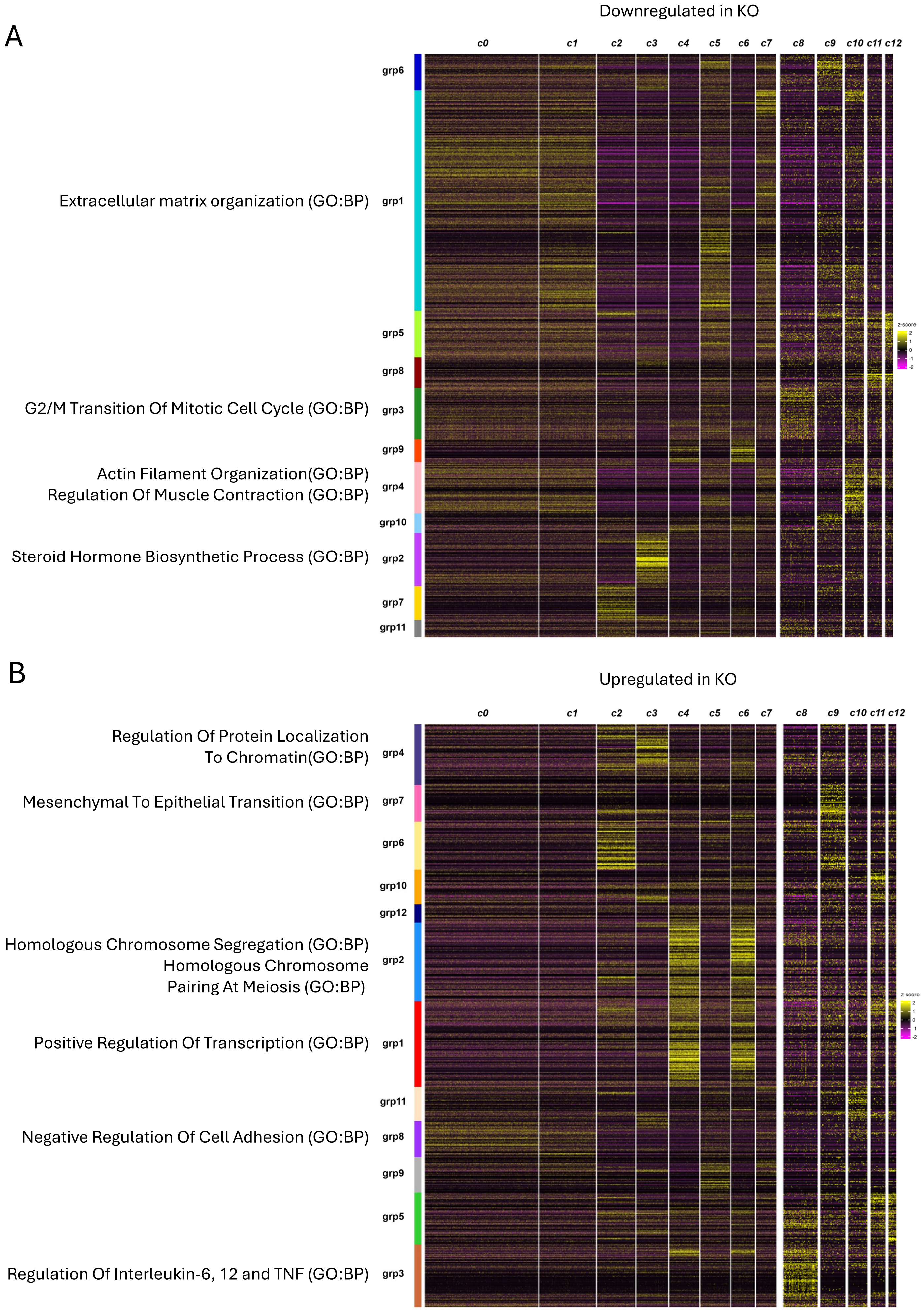

Supplementary Fig. 5: Bulk RNA-seq empirical projection using single-nuclei RNA-seq data. Bulk RNA-seq empirical projection was performed using our E14.5 testicular single-nuclei data. Heatmaps were generated using the average expression for each cluster of the downregulated (A) or upregulated (B) genes in *Nr2f2* knockout. Genes were then hierarchical grouped (grp#) based on the expression patterns across the 12 clusters. Gene ontology (biological process, GO:BP) analysis was performed on each of the different gene groups and the most representative statistically significant (adj P value<0.05) is presented next to each gene group.

Supplementary Figure 6

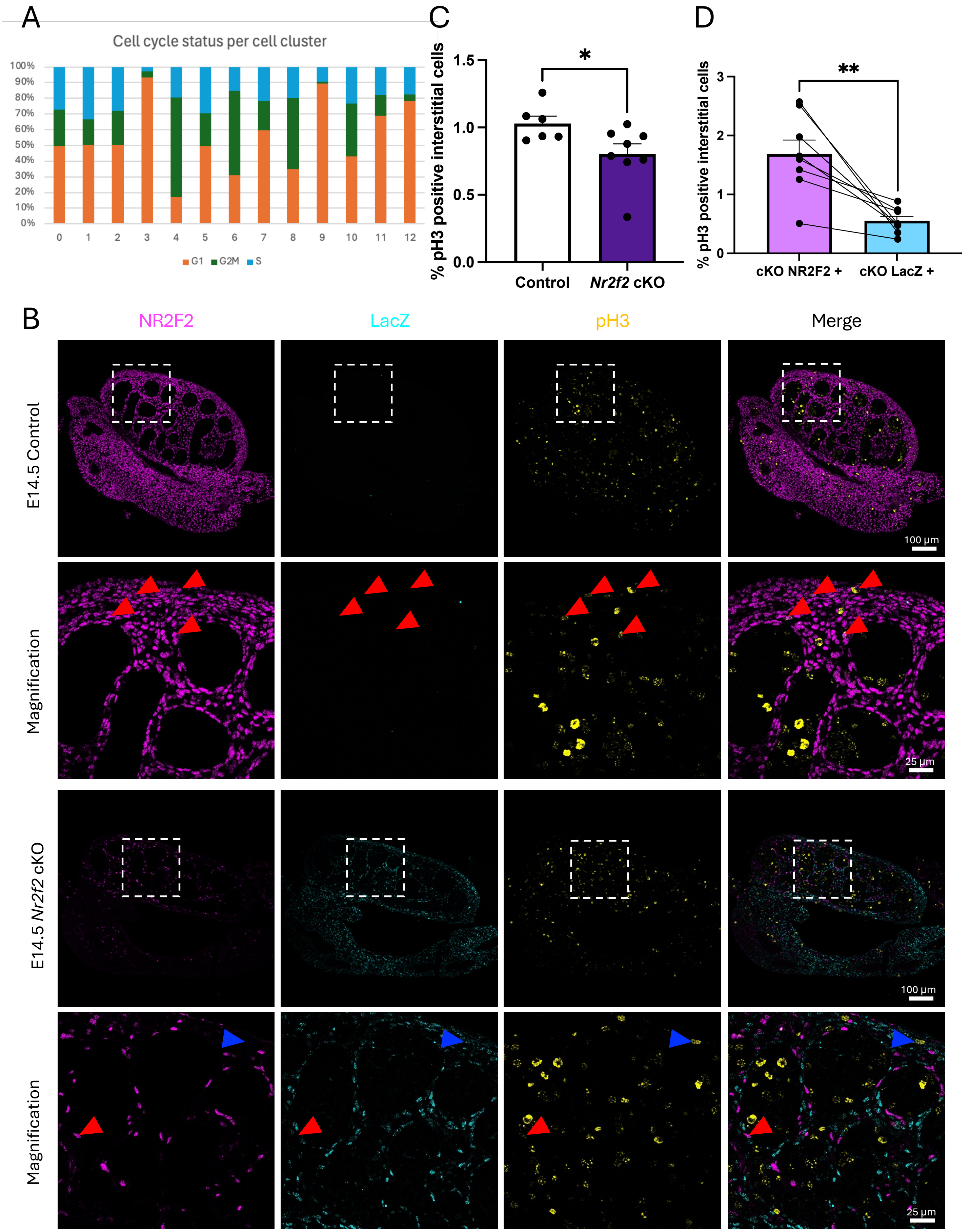

Supplementary Fig. 6: Proliferation analysis in Nr2f2 cKO gonads. (A) Cell cycle status (G1, G2M and S) of each cell per single-nuclei multiomic cluster, represented as a % of the total number of cells per each cluster. (B) Immunofluorescence for NR2F2 (magenta), LacZ (cyan) and pH3 (yellow) on E14.5 control or *Nr2f2* cKO testes. White dashed box indicates the magnified area. Red arrowheads indicate pH3+ NR2F2 positive/LacZ negative cells. Blue arrowheads indicate pH3+ NR2F2 negative/LacZ positive cells. (C) Quantification of the % of pH3 positive interstitial cells. Bars represent mean±s.e.m., n=6 control and n=8 *Nr2f2* cKO. Unpaired two-tailed t-test. *P<0.05. (D) Quantification of the % of pH3 positive interstitial cells NR2F2 positive or LacZ positive in *Nr2f2* cKO gonads. Bars represent mean±s.e.m., n=8 *Nr2f2* cKO. Paired two-tailed t-test. **P<0.01.

Supplementary Figure 7

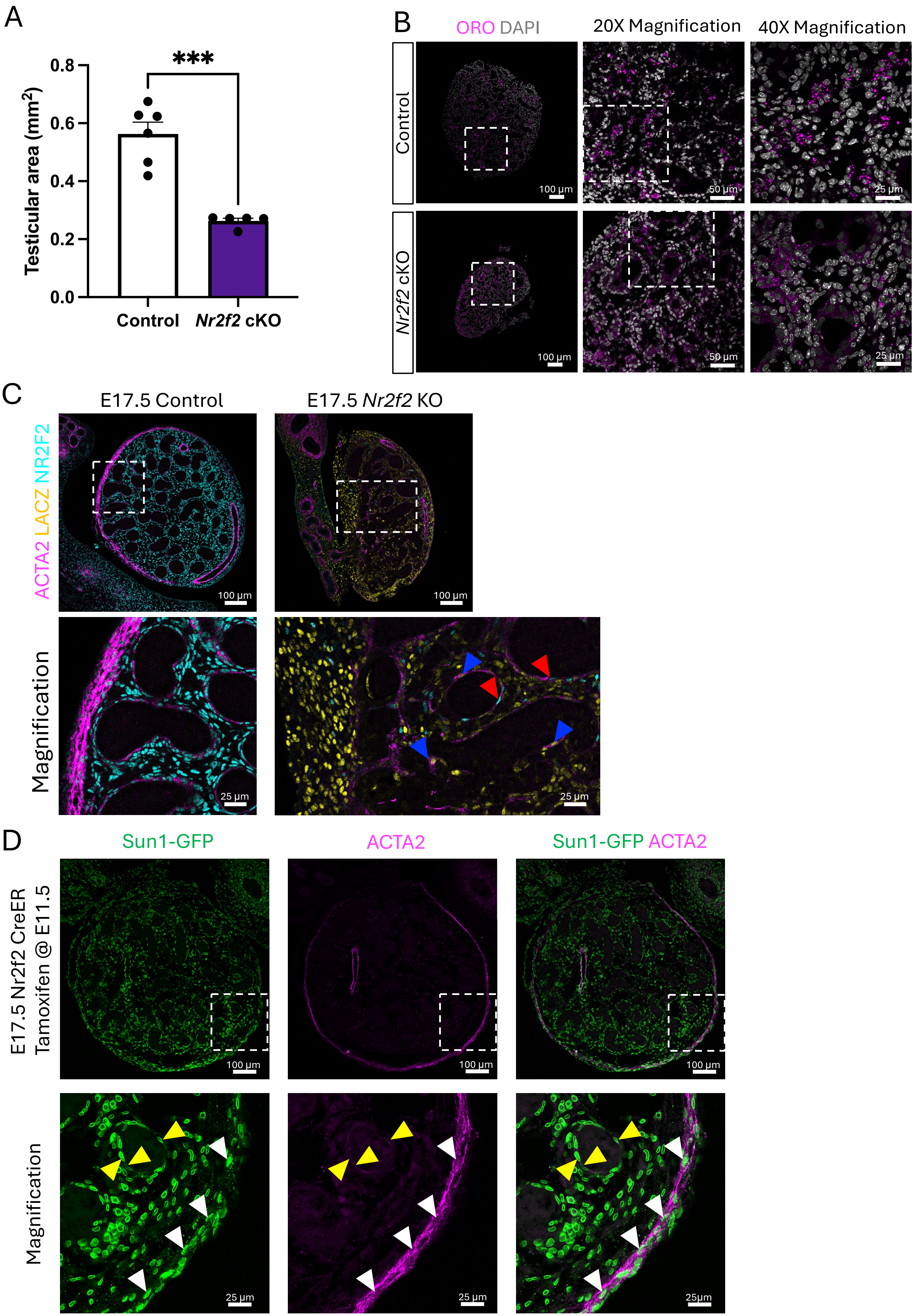

Supplementary Fig. 7: Nr2f2 positive interstitial cells give rise to Tunica and Peritubular myoid cells. (A) Quantification of the testicular area in E17.5 control vs *Nr2f2* conditional knockout gonads. Bars represent mean±s.e.m., n=6 control and n=5 *Nr2f2* cKO. Unpaired two-tailed t-test. ***P<0.001. (B) Oil red O (ORO, Magenta) staining in E17.5 control and *Nr2f2* cKO testes. Samples were counterstained with DAPI (grey). White dashed box indicates the magnified area. (C) Immunofluorescence of E17.5 control or *Nr2f2* cKO testes for ACTA2 (magenta), LACZ (Yellow) and NR2F2 (cyan). White dashed box indicates the magnified area. Red arrowheads indicate SMA+ NR2F2 positive/LacZ negative cells. Blue arrowheads indicate SMA+ NR2F2 negative/LacZ positive cells. (D) Lineage tracing experiment of the fetal testis-derived *Nr2f2*+ cells in the *Nr2f2*-CreER; *CAG-Sun1/sfGFP* embryos. Cre activity was induced by tamoxifen administration at E11.5 and immunofluorescence was performed on E17.5 testes, for GFP (Green) or ACTA2 (Magenta). White dashed box indicates the magnified area. Yellow arrowheads indicate SMA+ GFP positive PMCs. White arrowheads indicate SMA+ GFP positive tunica cells.

Supplementary Figure 8

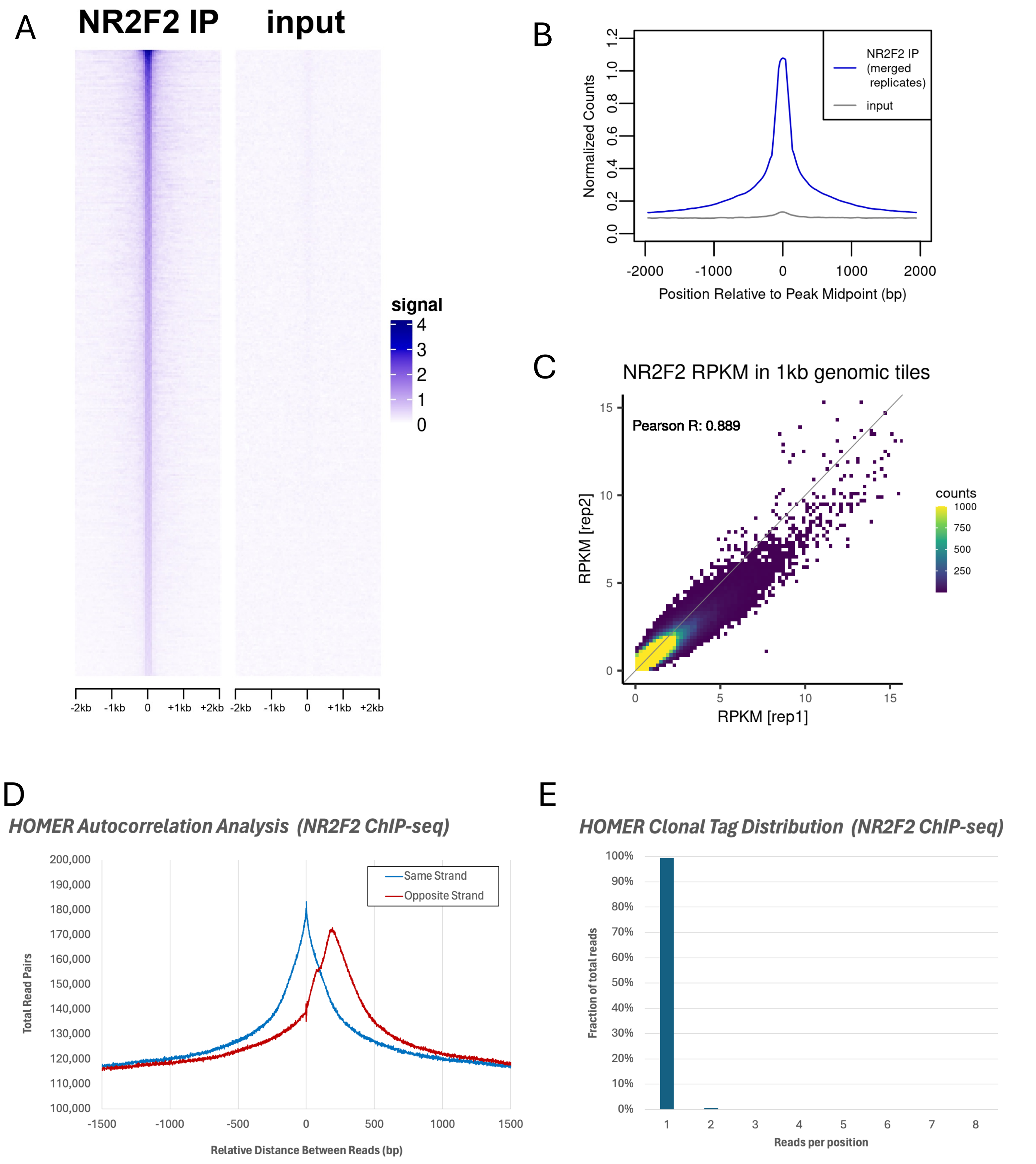

Supplementary Fig. 8: Summary QC for Nr2f2 ChIP-seq data. (A) Heatmap view of IP and input signal intensity in the 2kb regions flanking called peaks [N=10,069]. Mapped read counts are collected in 20-bp bins and normalized to 10 million uniquely-mapped reads. (B) Metaplot representation of the data in S9A. (C) Density scatterplot illustrating genome-wide similarity of the two Nr2f2 ChIP-seq biological replicates. RPKM = Reads Per Kilobase Per Million Mapped Reads. (D) Fragment autocorrelation plot indicating successful ChIP-seq samples (merged IP replicates) as evaluated by the HOMER makeTagDirectory function. (E) Very low clonality of post-processed ChIP-seq samples (merged IP replicates) as evaluated by the HOMER makeTagDirectory function.

Supplementary Figure 9

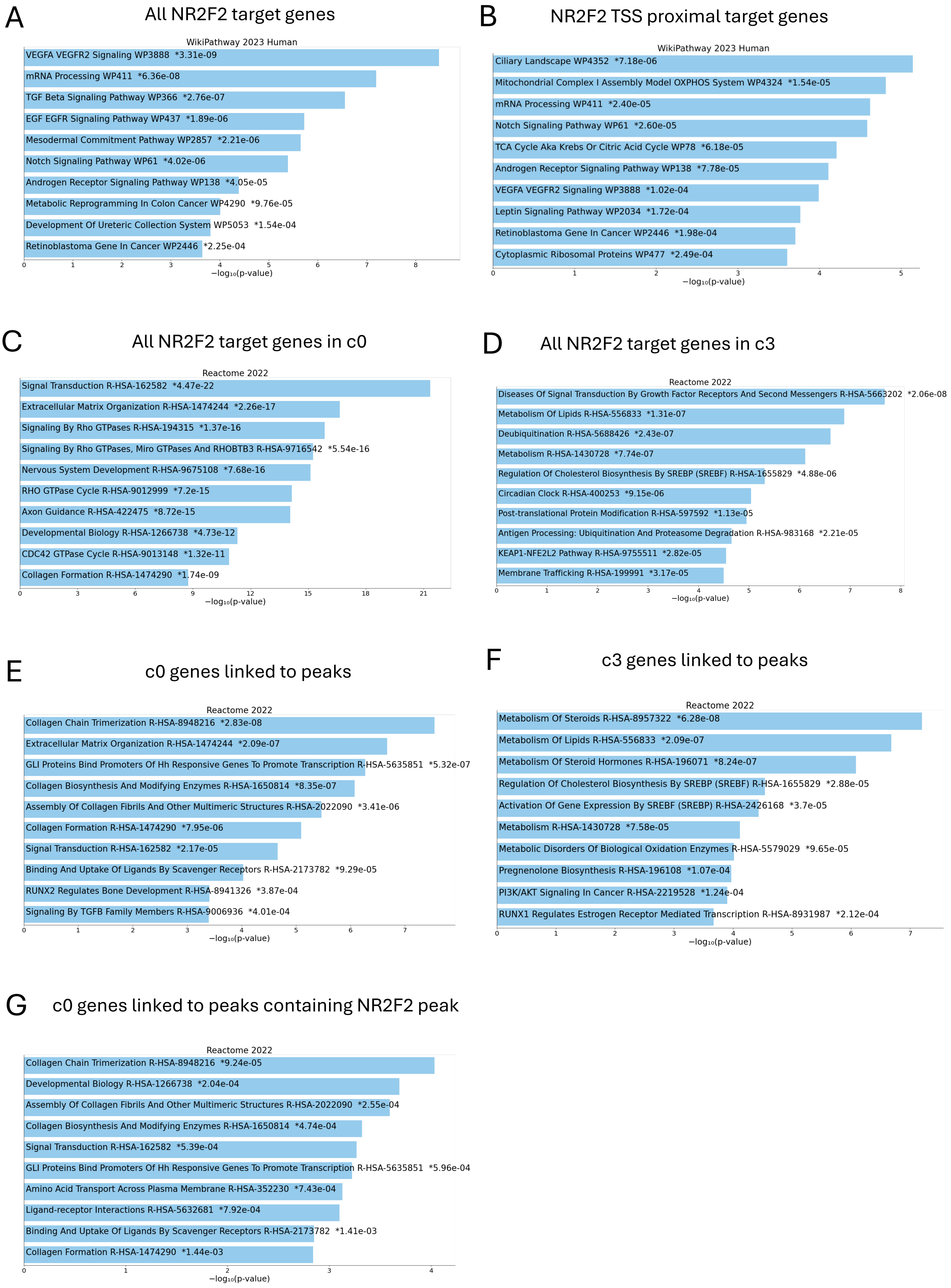

Supplementary Fig. 9: Pathway analysis of the ChIP-seq and ATAC-seq data. Pathway analysis (WikiPathway 2023) of all NR2F2 target genes (A) or all NR2F2 TSS proximal target genes (B). Pathway analysis (Reactome 2022) of all C0 (C) or C3 (D) DEGs with a significant NR2F2 peak associated. Pathway analysis (Reactome 2022) of all the linked genes to a differentially accessible peak in c0 (E) or c3 (F). (G) Pathway analysis (Reactome 2022) of all differentially accessible peaks on c0 vs c3, containing an NR2F2 ChIP-seq peak. * Indicates adjusted p-value <0.05.

Supplementary Figure 10

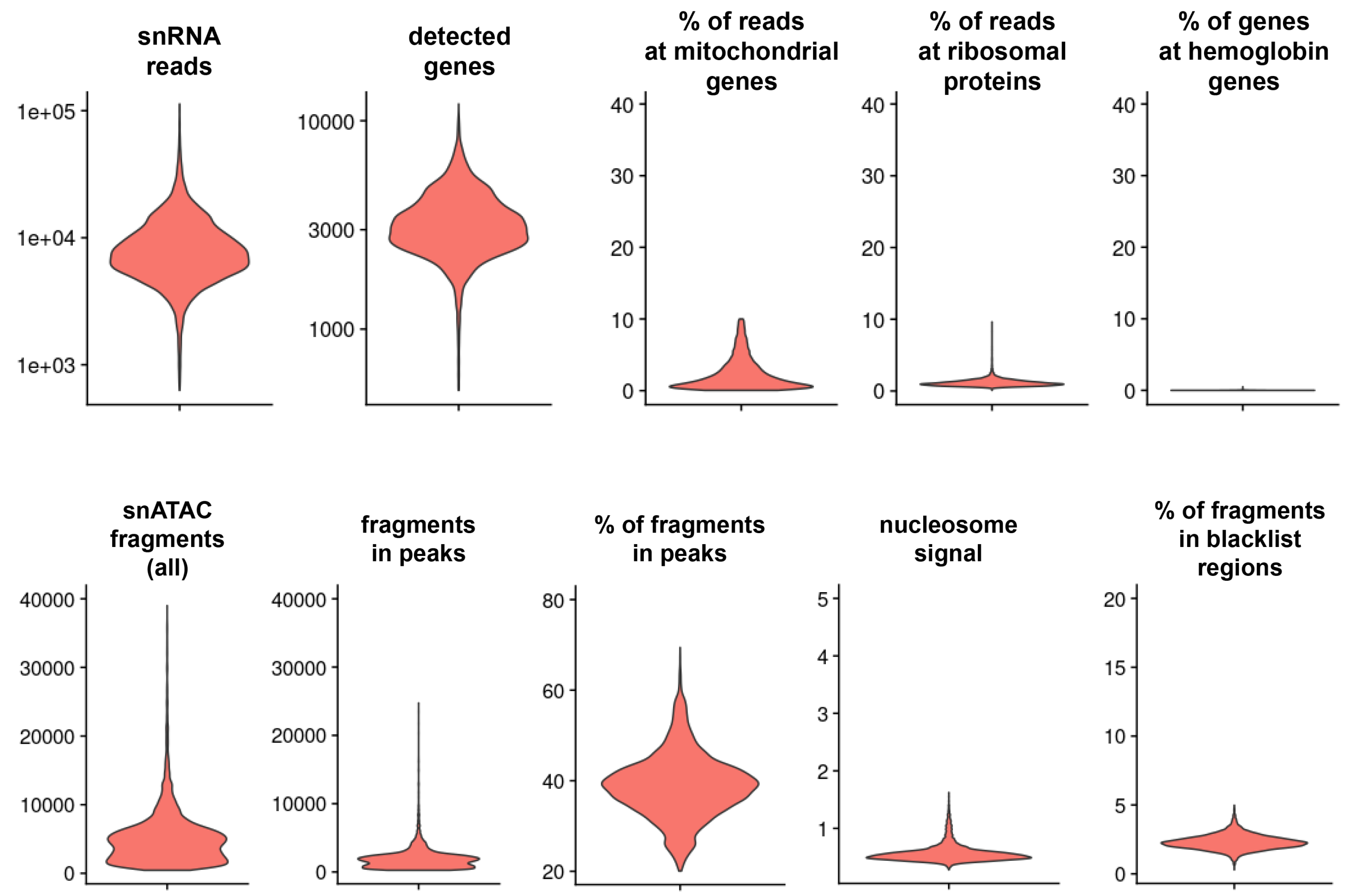

Supplementary Fig. 10: Summary QC for single-nucleus multiomics data. Violin plots illustrating per-cell distribution for standard QC metrics of the snRNA-seq assay (top row) and snATAC-seq assay (bottom row); only cells passing all QC filtering are included in these summary plots.
