# Supplementary material for: NR2F2 regulation of interstitial cell fate in the embryonic mouse testis and its impact on differences of sex development
Source: Nat Commun. 2025 Apr 29;16:3987. doi: 10.1038/s41467-025-59183-6 (PMC12038043; doi:10.1038/s41467-025-59183-6)
Supplement: Supplementary file 2 — Description of Addtional Supplementary Files [file 41467_2025_59183_MOESM2_ESM.pdf]

Description of Additional Supplementary Files

Supplementary Data 1. Differentially expressed genes (adjusted $p<0.05$) per single-nuclei multiomic cluster from E14.5 testes.

Supplementary Data 2. Differentially expressed genes (adjusted $p<0.05$) between c0 (interstitial cells) and c3 (leydig cells). This table also contains the pathway analysis (Reactome 2022) for c0 and c3 DEGs (adjusted $p<0.05$).

Supplementary Data 3. Bulk RNA-seq data from E14.5 control and Nr2f2 conditional knockout testes. This table contains QC summary, fragment size plot, fragment size data and the differential expression analysis (adjusted $p<0.05$). This table contains the affected population score, gene ontology analysis (biological process) for each of the different gene groups in the upregulated and downregulated datasets (adjusted $p<0.05$).

Supplementary Data 4. List of genes per gene group from the bulk RNA-seq empirical projection of the upregulated and downregulated DEGs (adjusted $p<0.05$) between E14.5 Nr2f2 cKO and control testes. This table contains gene ontology analysis (biological process) for each of the different gene groups in the upregulated and downregulated datasets (adjusted $p<0.05$).

Supplementary Data 5. Statistically significant NR2F2 peaks (fdr<1-5) called in E14.5 testes. This table includes the genomic context, the closest gene and the distance in bp to that gene. This table also contains the pathway analysis (Wikipathway 2023) for all or (Wikipathway 2023) for all or TSS proximal NR2F2 ChIP-seq targets (fdr<1-5) and for all the NR2F2 ChIP-seq targets (fdr<1-5) that are c0 and c3 DEGs (adjusted $p<0.05$) (Reactome 2022).

Supplementary Data 6. Differentially accessible peaks (adjusted $p<0.05$) between c0 (interstitial cells) and c3 (Leydig cells). This table includes the ATAC peak range, the ATAC peak, the distance to the nearest NR2F2 ChIP-seq peak and the linked genes to the peak. This table also contains the pathway analysis (Reactome 2022) for all the genes linked to c0 and c3 specific peaks, and the genes linked to c0 specific peaks that also contains a NR2F2 ChIP-seq binding peak (adjusted $p<0.05$).

Supplementary Data 7. Motifs enriched in differentially accessible peaks in c0 and c3. This table includes the list of enriched motifs for c0 and c3 (adjusted $p<0.05$), the DEGs analysis of the motifs in c0 and c3 (adjusted $p<0.05$) and the combination analysis with the Nr2f2 knockout RNA-seq and the NR2F2 ChIPseq. This table also contains the Motifs enriched in differentially accessible peaks in c0 vs c3 that that also contains a NR2F2 ChIP-seq binding peak.

Supplementary Data 8. Primary and secondary antibodies used for immunofluorescence.
