# Supplementary material for: NR2F2 regulation of interstitial cell fate in the embryonic mouse testis and its impact on differences of sex development
Source: Nat Commun. 2025 Apr 29;16:3987. doi: 10.1038/s41467-025-59183-6 (PMC12038043; doi:10.1038/s41467-025-59183-6)
Supplement: Supplementary file 11 — Reporting Summary [file 41467_2025_59183_MOESM11_ESM.pdf]

nature portfolio

Corresponding author(s): Humphrey Yao

Last updated by author(s): Mar 22, 2024

Reporting Summary

Nature Portfolio wishes to improve the reproducibility of the work that we publish. This form provides structure for consistency and transparency in reporting. For further information on Nature Portfolio policies, see our Editorial Policies and the Editorial Policy Checklist.

Statistics

For all statistical analyses, confirm that the following items are present in the figure legend, table legend, main text, or Methods section.

n/a	Confirmed	
☐	☒	The exact sample size (*n*) for each experimental group/condition, given as a discrete number and unit of measurement
☐	☒	A statement on whether measurements were taken from distinct samples or whether the same sample was measured repeatedly
☐	☒	The statistical test(s) used AND whether they are one- or two-sided<br>*Only common tests should be described solely by name; describe more complex techniques in the Methods section.*
☐	☒	A description of all covariates tested
☐	☒	A description of any assumptions or corrections, such as tests of normality and adjustment for multiple comparisons
☐	☒	A full description of the statistical parameters including central tendency (e.g. means) or other basic estimates (e.g. regression coefficient) AND variation (e.g. standard deviation) or associated estimates of uncertainty (e.g. confidence intervals)
☐	☒	For null hypothesis testing, the test statistic (e.g. *F*, *t*, *r*) with confidence intervals, effect sizes, degrees of freedom and *P* value noted<br>*Give P values as exact values whenever suitable.*
☐	☒	For Bayesian analysis, information on the choice of priors and Markov chain Monte Carlo settings
☐	☒	For hierarchical and complex designs, identification of the appropriate level for tests and full reporting of outcomes
☐	☒	Estimates of effect sizes (e.g. Cohen's *d*, Pearson's *r*), indicating how they were calculated

Our web collection on statistics for biologists contains articles on many of the points above.

Software and code

Policy information about availability of computer code

Data collection	Illumina NovaSeq 6000 instrument. Illumina NextSeq 500 instrument. a TapeStation 4200, QubitFlex Fluorometer, Zeiss LSM 900 confocal microscope using Zen software, Keyence Bz-X810 microscope,
Data analysis	10x Genomics Cell Ranger ARC v2.0.170, 71 scDblFinder v1.16.072 DecontX function of the celda package v1.18.173. Seurat v5.0.174 and Signac v1.12.075 Seurat FindMultiModalNeighbors function. Seurat FindClusters at resolution 0.3. Seurat FindMarkers Seurat DimPlot, FeaturePlot, and DotPlot functions. Seurat CellCycleScoring function. Monocle3 FindVariableFeatures function in Seurat; the hclust R MACS2 v2.2.9.177. Signac LinkPeaks function. Seurat FindMarkers fSignac FindMotifs, JASPAR202078 TFBSTools79 getMatrixSet. R v4.3.2. TrimGalore v0.6.782, Bowtie v2.1.083, MarkDuplicates.jar (Picard tool suite v1.11084), HOMER v4.11.185 'findPeaks' function, HOMER v4.11.1 'findMotifsGenome.pl'<br>TrimGalore v0.6.782. (Subread v1.5.0-p1), DESeq2 v1.28.188, Enrichr, deepvenn.<br>ComplexHeatmap v2.18.093., hclust, dendextend v1.17.194, Seurat's ScaleData function.

For manuscripts utilizing custom algorithms or software that are central to the research but not yet described in published literature, software must be made available to editors and reviewers. We strongly encourage code deposition in a community repository (e.g. GitHub). See the Nature Portfolio guidelines for submitting code & software for further information.

Data

Policy information about availability of data

All manuscripts must include a data availability statement. This statement should provide the following information, where applicable:

- Accession codes, unique identifiers, or web links for publicly available datasets
- A description of any restrictions on data availability
- For clinical datasets or third party data, please ensure that the statement adheres to our policy

Single-nucleus multiomic data have been deposited at GEO: GSE275901. NR2F2 ChIP-seq data have been deposited at GEO: GSE275900. Nr2f2 cKO Bulk RNA-seq data have been deposited at GEO: GSE275902. Data are publicly available as of the date of publication. Source data are provided with this paper. Other requests for data may be directed to and will be fulfilled by the Lead Contact, Humphrey Yao.

Research involving human participants, their data, or biological material

Policy information about studies with human participants or human data. See also policy information about sex, gender (identity/presentation), and sexual orientation and race, ethnicity and racism.

Reporting on sex and gender	*Use the terms sex (biological attribute) and gender (shaped by social and cultural circumstances) carefully in order to avoid confusing both terms. Indicate if findings apply to only one sex or gender; describe whether sex and gender were considered in study design; whether sex and/or gender was determined based on self-reporting or assigned and methods used. Provide in the source data disaggregated sex and gender data, where this information has been collected, and if consent has been obtained for sharing of individual-level data; provide overall numbers in this Reporting Summary. Please state if this information has not been collected. Report sex- and gender-based analyses where performed, justify reasons for lack of sex- and gender-based analysis.*
Reporting on race, ethnicity, or other socially relevant groupings	*Please specify the socially constructed or socially relevant categorization variable(s) used in your manuscript and explain why they were used. Please note that such variables should not be used as proxies for other socially constructed/relevant variables (for example, race or ethnicity should not be used as a proxy for socioeconomic status). Provide clear definitions of the relevant terms used, how they were provided (by the participants/respondents, the researchers, or third parties), and the method(s) used to classify people into the different categories (e.g. self-report, census or administrative data, social media data, etc.) Please provide details about how you controlled for confounding variables in your analyses.*
Population characteristics	*Describe the covariate-relevant population characteristics of the human research participants (e.g. age, genotypic information, past and current diagnosis and treatment categories). If you filled out the behavioural & social sciences study design questions and have nothing to add here, write "See above."*
Recruitment	*Describe how participants were recruited. Outline any potential self-selection bias or other biases that may be present and how these are likely to impact results.*
Ethics oversight	*Identify the organization(s) that approved the study protocol.*

Note that full information on the approval of the study protocol must also be provided in the manuscript.

Field-specific reporting

Please select the one below that is the best fit for your research. If you are not sure, read the appropriate sections before making your selection.

☒ Life sciences ☐ Behavioural & social sciences ☐ Ecological, evolutionary & environmental sciences

For a reference copy of the document with all sections, see nature.com/documents/nr-reporting-summary-flat.pdf

Life sciences study design

All studies must disclose on these points even when the disclosure is negative.

Sample size	More or equal than 4, less or equal than 10
Data exclusions	*Describe any data exclusions. If no data were excluded from the analyses, state so OR if data were excluded, describe the exclusions and the rationale behind them, indicating whether exclusion criteria were pre-established.*
Replication	Performed in at least 3 replicates
Randomization	Samples were randomly collected and analyzed.
Blinding	Phenotyping was performed blindly

Reporting for specific materials, systems and methods

We require information from authors about some types of materials, experimental systems and methods used in many studies. Here, indicate whether each material, system or method listed is relevant to your study. If you are not sure if a list item applies to your research, read the appropriate section before selecting a response.

Materials & experimental systems

n/a	Involved in the study	
☐	☒	Antibodies
☒	☐	Eukaryotic cell lines
☒	☐	Palaeontology and archaeology
☐	☒	Animals and other organisms
☒	☐	Clinical data
☒	☐	Dual use research of concern
☒	☐	Plants

Methods

n/a	Involved in the study	
☐	☒	ChIP-seq
☒	☐	Flow cytometry
☒	☐	MRI-based neuroimaging

Antibodies

Antibodies used

Antibody type Conjucated Host Target Catalog Company Dilution
Primary No Rabbit p-Histone H3 Antibody (Ser 10) sc-8656-R Santa Cruz 1:200
Primary No Rabbit ALDH1A2 HPA010022 Sigma-Aldrich 1:200
Primary No Mouse ITGA8 sc-365798 Santa Cruz 1:200
Primary No Mouse LGI1 MA5-27652 Invitrogen 1:200
Primary No Mouse NR2F2 PP-H7147-00 R&D Systems 1:200
Primary No Rat NR5A1 KO610 Cosmo Bio 1:200
Primary No Goat AMH sc-6886 Santa Cruz 1:500
Primary No Goat CYP17A1 sc-46081 Santa Cruz 1:200
Primary No Rabbit ARX Collaborator DOI: 10.1038/ng1009 1:200
Primary No Rabbit PDGFRA ab203491 Abcam 1:200
Primary No Rabbit SMA ab5694 Abcam 1:200
Primary No Goat PECAM1 AF3628 R&D Systems 1:200
Primary No Rabbit NR2F2 ab211777 Abcam 1:200
Primary No Goat GFP ab5450 Abcam 1:200
Primary No Chicken GFP ab13970 Abcam 1:200
Primary No Rabbit HSD3B1 K0607 CosmoBio 1:200
Primary No Chicken LACZ ab9361 Abcam 1:1000
Primary No Rat TRA98 73-003 DiagnoCine 1:1000
Primary No Rabbit MAFB A700-046 Bethyl Labs 1:200
Secondary Alexa Fluor 488 Donkey Mouse IgG A21202 Invitrogen 1:200
Secondary Alexa Fluor 488 Donkey Rabbit A21206 Invitrogen 1:200
Secondary Alexa Fluor 488 Donkey Rat A21208 Invitrogen 1:200
Secondary Alexa Fluor 488 Donkey Goat A11055 Invitrogen 1:200
Secondary Alexa Fluor 568 Donkey Mouse IgG A10037 Invitrogen 1:200
Secondary Alexa Fluor 568 Donkey Rabbit A10042 Invitrogen 1:200
Secondary Alexa Fluor 568 Donkey Rat A78946 Invitrogen 1:200
Secondary Alexa Fluor 568 Donkey Goat A11057 Invitrogen 1:200
Secondary Alexa Fluor 647 Donkey Mouse IgG A31571 Invitrogen 1:200
Secondary Alexa Fluor 647 Donkey Rabbit A31573 Invitrogen 1:200
Secondary DyLight™ 650 Donkey Rat SA510029 Invitrogen 1:200
Secondary Alexa Fluor 647 Donkey Goat A21447 Invitrogen 1:200
Secondary Cy5 Donkey Chicken 703-175-155 Jackson 1:200

Validation

Validations were performed by IF in samples known to express the protein of interest.

Animals and other research organisms

Policy information about studies involving animals; ARRIVE guidelines recommended for reporting animal research, and Sex and Gender in Research

Laboratory animals

Mice were housed on a 12 hr light:dark cycle, temperature range 21–23°C, and relative humidity range from 40% to 50%. Nr5a1-cre mice (B6D2-Tg(Nr5a1-cre)2Klp) were provided by the Dr. Keith Parker68. Nr2f2f/f (B6;129S7-Nr2f2<tm2Tsa>/Mmmh) mice were provided by Dr. Sofia Tsai39. Nr2f2-CreER mice (B6.129S-Nr2f2<tm1(icre/ERT2)Hy) were generated by NIEHS gene editing and mouse model core facility69. Rosa-tdTomato (B6.Cg-Gt(ROSA)26Sor<tm9(CAG-tdTomato)Hze>/J), CAG-Sun1/sfGFP (B6;129-Gt(ROSA)26Sor<tm5(CAG-Sun1/sfGFP)Nat>/J) and Wt1-CreER (Wt1<tm2(cre/ERT2)Wtp>/J) mice were purchased from Jackson Laboratory (stock number 007909, 021039 and 010912, respectively). CD-1 (Charles River stock number 022)

Wild animals

Provide details on animals observed in or captured in the field; report species and age where possible. Describe how animals were

Wild animals	*caught and transported and what happened to captive animals after the study (if killed, explain why and describe method; if released, say where and when) OR state that the study did not involve wild animals.*
Reporting on sex	Yes, only males were used as we are studying testicular morphogenesis
Field-collected samples	*For laboratory work with field-collected samples, describe all relevant parameters such as housing, maintenance, temperature, photoperiod and end-of-experiment protocol OR state that the study did not involve samples collected from the field.*
Ethics oversight	All animal procedures were approved by the National Institute of Environmental Health Sciences (NIEHS) Animal Care and Use Committee and are in compliance with a NIEHS-approved animal study proposal (2010-0016).

Note that full information on the approval of the study protocol must also be provided in the manuscript.

Plants

Seed stocks	*Report on the source of all seed stocks or other plant material used. If applicable, state the seed stock centre and catalogue number. If plant specimens were collected from the field, describe the collection location, date and sampling procedures.*
Novel plant genotypes	*Describe the methods by which all novel plant genotypes were produced. This includes those generated by transgenic approaches, gene editing, chemical/radiation-based mutagenesis and hybridization. For transgenic lines, describe the transformation method, the number of independent lines analyzed and the generation upon which experiments were performed. For gene-edited lines, describe the editor used, the endogenous sequence targeted for editing, the targeting guide RNA sequence (if applicable) and how the editor was applied.*
Authentication	*Describe any authentication procedures for each seed stock used or novel genotype generated. Describe any experiments used to assess the effect of a mutation and, where applicable, how potential secondary effects (e.g. second site T-DNA insertions, mosiacism, off-target gene editing) were examined.*

ChIP-seq

Data deposition

☒ Confirm that both raw and final processed data have been deposited in a public database such as GEO.

☒ Confirm that you have deposited or provided access to graph files (e.g. BED files) for the called peaks.

Data access links<br>*May remain private before publication.*	GSE275900
Files in database submission	*Provide a list of all files available in the database submission.*
Genome browser session<br>(e.g. UCSC)	*Provide a link to an anonymized genome browser session for "Initial submission" and "Revised version" documents only, to enable peer review. Write "no longer applicable" for "Final submission" documents.*

Methodology

Replicates	2 biological replicates
Sequencing depth	*Describe the sequencing depth for each experiment, providing the total number of reads, uniquely mapped reads, length of reads and whether they were paired- or single-end.*
Antibodies	Active Motif, CA 61213
Peak calling parameters	calling peaks was performed by the HOMER v4.11.185 'findPeaks' function with parameters "-style factor -fdr 1e-5". Peak regions were subsequently re-sized to a width of 300bp centered on the called peak midpoints. Peaks overlapping with mm10 blacklist regions were excluded from downstream analysis.
Data quality	In Supplementary Figure 8
Software	*Describe the software used to collect and analyze the ChIP-seq data. For custom code that has been deposited into a community repository, provide accession details.*
